# Development and application of an amplified luminescent proximity homogeneous assay-linked immunosorbent assay for the accurate quantification of kidney injury molecule-1

**DOI:** 10.3389/fmolb.2023.1280681

**Published:** 2024-01-18

**Authors:** Yulin Fu, Danqin Sun, Yuan Qin, Tianyu Zheng, Zixuan Zhou, Xiumei Zhou, Xueqin Zhao, Yan Xu, Biao Huang

**Affiliations:** ^1^ College of Life Sciences and Medicine, Zhejiang Sci-Tech University, Hangzhou, China; ^2^ Department of Nephrology, Jiangnan University Medicine Center, Wuxi, China; ^3^ Department of Nephrology, Suzhou Ninth People’s Hospital, Suzhou Ninth Hospital Affiliated to Soochow University, Suzhou, China

**Keywords:** kidney injury molecule-1, amplified luminescent proximity homogeneous assay linked immunosorbent assay, double-antibody sandwich, serum, urine, acute kidney injury

## Abstract

**Background:** Kidney injury molecule-1 (Kim-1), a specific marker of kidney injury, is usually not expressed in normal kidneys or at very low levels but is highly expressed in injured renal tubular epithelial cells until the damaged cells recover completely. Therefore, we aimed to develop an efficient and highly sensitive assay to accurately quantify Kim-1 levels in human serum and urine.

**Methods:** In this study, a novel immunoassay was developed and named amplified luminescent proximity homogeneous assay-linked immunosorbent assay (AlphaLISA). Anti-Kim-1 antibodies can be directly coupled to carboxyl-modified donor and acceptor beads for the rapid detection of Kim-1 by double-antibody sandwich method. Serum and urine samples for Kim-1 measurements were obtained from 129 patients with nephropathy and 17 healthy individuals.

**Results:** The linear range of Kim-1 detected by AlphaLISA was 3.83–5000 pg/mL, the coefficients of variation of intra-assay and inter-assay batches were 3.36%–4.71% and 5.61%–11.84%, respectively, and the recovery rate was 92.31%–99.58%. No cross reactions with neutrophil gelatinase-associated lipocalin, liver-type fatty acid binding protein, and matrix metalloproteinase-3 were observed. A good correlation (*R* ^2^ = 0.9086) was found between the findings of Kim-1-TRFIA and Kim-AlphaLISA for the same set of samples. In clinical trials, both serum and urine Kim-1 levels were significantly higher in patients with nephropathy than in healthy individuals, especially in patients with acute kidney injury. Furthermore, serum Kim-1 was superior to urinary Kim-1 in distinguishing between patients with nephropathy and healthy individuals.

**Conclusion:** The developed Kim-1-AlphaLISA is highly efficient, precise, and sensitive, and it is suitable for the rapid detection of patients with acute kidney injury.

## 1 Introduction

Kidney injury molecule-1 (Kim-1) is a type I transmembrane protein that plays a key role in renal tubulointerstitial injury; it is also known as T cell immunoglobulin mucin-1 (Tim-1) (Ichimura et al., 1998). The expression of this protein is upregulated in proximal tubular cells of injured kidneys, and its extracellular structural domain is cleaved and released (Bailly et al., 2002). Kim-1 functions as a phosphatidylserine receptor that recognizes apoptotic cells and guides them to lysosomes. It also functions as an acceptor for oxidized lipoproteins and is adept in receiving “eat me” signals from apoptotic cells. Kim-1 is the first known molecule that transforms kidney proximal epithelial cells into semi-specialized phagocytes (Savill and Fadok, 2000; Ichimura et al., 2008). Kim-1 can modulate immune responses in kidney injury by promoting the clearance of dead cells from renal tubular cells. The phagocytosis of apoptotic cells downregulates pro-inflammatory immune responses. Moreover, Kim-1 has been reported to be involved in renal interstitial fibrosis and inflammation in kidney disease (Peters et al., 2011). Thus, Kim-1 plays an important role in kidney disease.

Kim-1 is a promising biomarker of kidney injury. Kim-1 level corresponds to the severity of kidney injury, and various kidney diseases are associated with tubular injury, such as acute kidney injury (AKI) (Bellomo et al., 2012), lupus nephritis (LN) (Lan-Ting et al., 2020), diabetic nephropathy (DN) (Liu et al., 2023), membranous nephropathy (MN) (Huang et al., 2021), and IgA nephropathy (IgAN) (Lai et al., 2005). Currently, the diagnosis of AKI is based primarily on serum creatinine levels; however, changes in creatinine levels occur later after renal injury, and the effects of non-renal factors can contribute to elevated creatinine levels (Wajda et al., 2020). Elevation of Kim-1 may precede elevation of serum creatinine and has been shown to be sensitive to early kidney injury in human and animal subjects (Chiusolo et al., 2010; Vaidya et al., 2010). Compared with the general clinical indicators, Kim-1 has a more timely response to kidney injury; therefore, the application of Kim-1 detection in kidney disease has strong potential.

In clinical practice, enzyme-linked immunosorbent assay (ELISA), fluorescence immunochromatography assay, and electrochemiluminescence immunoassay are commonly utilized to detect the concentration of Kim-1 in blood or urine (Han et al., 2002; Chaturvedi et al., 2009; Vaidya et al., 2009; Zhang et al., 2013). In recent years, some novel detection techniques have been developed, such as time-resolved fluorescence immunoassay (TRFIA) based on double-antibody sandwich, to detect serum Kim-1 (Shaoxiong et al., 2022). However, these techniques have limitations, such as insufficient sensitivity or time-consuming process. By contrast, AlphaLISA has remarkable application potential.

**FIGURE 1:**
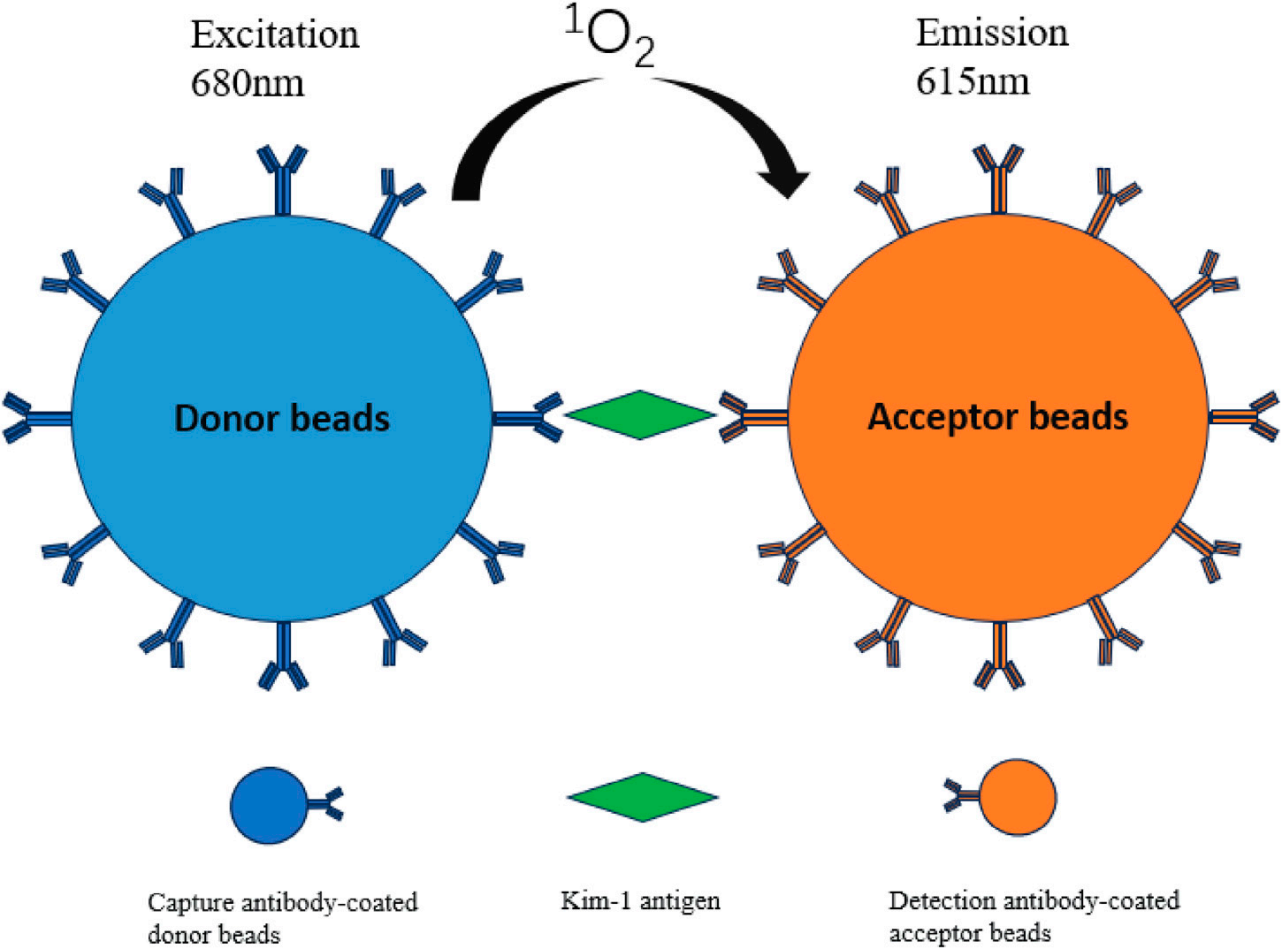
Principle of AlphaLISA for detecting kidney injury molecule-1.

AlphaLISA is a nanosphere-based homogeneous immunoassay characterized by rapid measurement, high sensitivity, and good repeatability. This method uses two types of nanobeads, namely, donor and acceptor beads. In recent years, carboxyl-modified donor and acceptor beads have been developed. Carboxyl-modified donor beads and acceptor beads can directly conjugate antibodies, thereby effectively simplifying the steps and shortening the reaction time. The donor and acceptor beads used in AlphaLISA are coated with photosensitizers and luminescent agent, respectively. The donor beads are coated with a photosensitizer (i.e., benzene dimethyl blue); under light excitation at 680 nm, oxygen in the environment near the surface of the donor beads is decomposed and converted into singlet oxygen, which migrates to the acceptor beads. The surface of the acceptor beads is coated with a luminescent agent, dimethylthiophene derivative, chelated with the rare earth atom europium; when the energy is finally transferred to the rare earth atom europium, the excitation light at 615 nm is excited (Xiang et al., 2022). The emission has high intensity, long life, and sharp peaks (Liu et al., 2013). A schematic demonstration of the method is shown in Figure 1.

In this research, we developed AlphaLISA based on carboxyl-modified microspheres for the rapid and precise detection of Kim-1 levels in serum and urine of patients with kidney disease.

## 2 Materials and methods

### 2.1 Reagents and instruments

The detection antibody, capture antibody, and antigen standard of Kim-1 were purchased from Shanghai Uning Biotechnology Co., Ltd. Donor microspheres, acceptor microspheres, and Kim-1 kit (TRFIA) were provided by Zhejiang Boshi Biological Technology Co., Ltd. A low-temperature and high-speed centrifuge was purchased from Heraeus (Germany). An electrically heated incubator was purchased from Jiangsu Taicang Jinghong Experimental Equipment Co., Ltd. The time-resolved fluorescence immunoassay analyzer and Tecan M200 for AlphaLISA were provided by Guangdong Foshan Daan Medical Equipment Co., Ltd. and TTecan Trading AG (Myron, Switzerland), respectively.

### 2.2 Buffer composition

The buffers used in this study were provided by Zhejiang Boshi Biological Technology Co., Ltd. and included coupling buffer (50 mmol/L MES, pH 6.0), blocking buffer (50 mmol/L Tris-HCl, 0.9% NaCl, 0.5% BSA, and 0.05% [v/v] Proclin300, pH 7.8), and standard dilution (50 mmol/L Tris-HCl, 0.9% NaCl, 0.2% BSA, and 0.05% Proclin300, pH 7.8).

### 2.3 Serum samples

Between July 2020 and September 2022, serum and urine samples were collected from nephropathic patients (129) and healthy volunteers (17) in the Department of Nephrology, Jiangnan University Medicine Center (Wuxi, China). Among them, patients with nephropathy could be categorized into five groups, namely, AKI (28), MN (32), DN (32), LN (11), and IgAN (26). Blood and urine were collected from each participant, separated at 4,000 rpm for 10 min, and stored at −20°C until experimental use. All participants gave informed consent before the study. The study was approved by the Medical Ethics Committee of Jiangnan University.

### 2.4 Establishment of Kim-1-AlphaLISA

#### 2.4.1 Purification of antibodies

An appropriate amount of Kim-1 capture antibody was added to a 50 KD ultrafiltration tube, which was centrifuged at 1,000 rpm for 5 min at 4°C. The filtrate was then discarded. About 300 μL of coupling buffer was added to the ultrafiltration tube, which was centrifuged at 1,000 rpm for 5 min at 4°C. The filtrate was then discarded. This process was repeated eight times. The ultrafiltration tube was then inverted and centrifuged at 3,000 rpm for 1 min. The tube was turned over and added with 50 µL of coupling buffer. The tube was left to stand for 2 min before being inverted and centrifuged again to collect the antibody, namely, the purified capture antibody. The purification steps for the detection antibody were the same as above.

#### 2.4.2 Coupling of microspheres to antibodies

The coupling buffer was added with 1 mg of donor microspheres for shock washing and centrifuged at 17,000 rpm for 20 min. After removal of the supernatant, the sample was sonicated and mixed by adding coupling buffer. Subsequently, the purified capture antibody was added, quickly mixed, and incubated for 2 h at room temperature with shaking and protected from light. After incubation, 100 µL of blocking buffer was added and incubated for 1 h at room temperature with shaking and protected from light. To remove the microspheres of unbound antibody, we centrifuged the microspheres at 1,100 rpm for 15 min, removed the supernatant, and continued to add coupling buffer to ultrasonically disperse the collection, namely, the donor microspheres coupled with the capture antibody. We then used the same method as described above to couple the detection antibody to the acceptor microspheres.

#### 2.4.3 Preparation of Kim-1 standard

Kim-1 antigen standard was diluted to the following concentrations by using standard diluent: 50, 500, 1,250, 2,500, and 5,000 pg/mL. In this work, 0 concentration standard was replaced with standard diluent. The standard products were stored at 4°C until use.

#### 2.4.4 Kim-1-AlphaLISA procedure

Each well of the 96-well microtiter plate was filled with 25 µL of the Kim-1 standard or samples (serum or urine [1:5 dilution]), followed by 50 µL each of donor microspheres coupled to the capture antibody and acceptor microspheres coupled to detect the antibody. Both microspheres were diluted to a suitable concentration with the standard dilution buffer. The samples were incubated for 20 min at 37°C and shielded from light, and the AlphaLISA signal was detected using Tecan M200.

### 2.5 Optimization and assessment of Kim-1-AlphaLISA

#### 2.5.1 Selection of the optimum donor beads concentration

The standard dilution solution was utilized to dilute the donor microspheres conjugated to capture antibodies to various concentrations (5, 10, 25, 50, 75, and 100 ng/mL), whereas the concentration of the acceptor microspheres conjugated to detecting antibodies was temporarily set at 75 ng/mL. The signal value of the high-concentration standard was detected.

#### 2.5.2 Selection of the optimum acceptor beads concentration

The acceptor microspheres conjugated to detecting antibodies were diluted to different concentrations (5, 10, 25, 50, 75, and 100 ng/mL) by using the standard dilution solution, whereas the donor microspheres conjugated to capture antibodies were temporarily set at 75 ng/mL. The standard signal value at high concentrations was determined.

#### 2.5.3 Selection of optimal incubation time

Optimal concentrations of donor beads and acceptor beads were selected and detected at incubation times of 5, 10, 15, 20, 25, 30, and 35 min for the 2,500 pg/mL Kim-1 standard.

#### 2.5.4 Standard curve

The standard curve equation was obtained with Graphpad prism software to create a graph using the standard concentration of KIM-1 as the *X*-axis and the AlphaLISA signals measuring different KIM-1 standard concentrations as the *Y*-axis. Linear analysis and basic linear regression were used to treat the standard curve.

#### 2.5.5 Sensitivity

The mean ( $\bar{X}$ ) and standard deviation (SD) of the fluorescence counting results were calculated after 10 measurements of the AlphaLISA signal for 0 concentration standards. The sensitivity is the concentration corresponding to the standard curve at $\bar{X}+2\text{SD}$ fluorescence values.

#### 2.5.6 Precision

Low, medium, and high concentrations of the Kim-1 antigen standard (500, 1,250, and 2,500 pg/mL, respectively) were used to evaluate intra-assay and inter-assay precision. For intra-assay precision, 10 tests using three antigen standards at varying concentrations were used to calculate the mean and standard deviation of AlphaLISA signals. The coefficient of variation in the experiment was calculated as $\text{SD}/\bar{X}$ of the test results. For inter-assay precision, the $\bar{X}$ and SD values of AlphaLISA signals were calculated by using three antigen standards at different concentrations 10 times a day for 3 days. The inter-assay coefficient of variation was calculated as $\text{SD}/\bar{X}$ .

#### 2.5.7 Specificity

As interferers, MMP-3 samples at 500 ng/mL, L-FABP samples at 100 ng/mL, and NGAL samples at 100 ng/mL were tested. According to the standard curve, the measured AlphaLISA signal indicated the exact concentration.

#### 2.5.8 Accuracy

Recovery rate was used to evaluate the accuracy of Kim-1-AlphaLISA. Three Kim-1 antigen standards (500, 2,500, and 5,000 pg/mL) were mixed with the serum sample with a known concentration of 2047.65 pg/mL in a 1:9 ratio. The actual concentration of the mixed solution was calculated from the standard curve based on the AlphaLISA signal measured in the mixed solution. Theoretical concentrations of 1892.89, 2092.89, and 2,342.89 pg/mL were prepared by mixing the serum and antigen standard in a proportional manner. The recovery rate (%) was calculated as follows: (measured concentration/theoretical concentration) × 100%. The recovery rate experiment was repeated three times.

#### 2.5.9 Comparison of the correlation between AlphaLISA and TRFIA methods

Plasma samples from 13 patients with kidney disease and 13 healthy individuals were co-tested using Kim-1-TRFIA and Kim-1-AlphaLISA, and the correlation between the two results was analyzed.

### 2.6 Clinical application of Kim-1-AlphaLISA

Serum and urine Kim-1 levels were analyzed by KIM-1-AlphaLISA in 28 patients with AKI, 32 patients with MN, 32 patients with DN, 11 patients with LN, 26 patients with IgAN, and 17 healthy subjects.

### 2.7 Statistical analysis

Data were statistically analyzed using SPSS 26.0 software, and graphs were created using GraphPad Prism 8.3. Spearman’s correlation analysis was used to assess the correlation between the two methods. Unpaired *t*-test was used to calculate the significance of differences in the values of the groups. Statistical significance was set at *p* < 0.05.

## 3 Results

### 3.1 Selection of the optimum donor beads concentration

**FIGURE 2:**
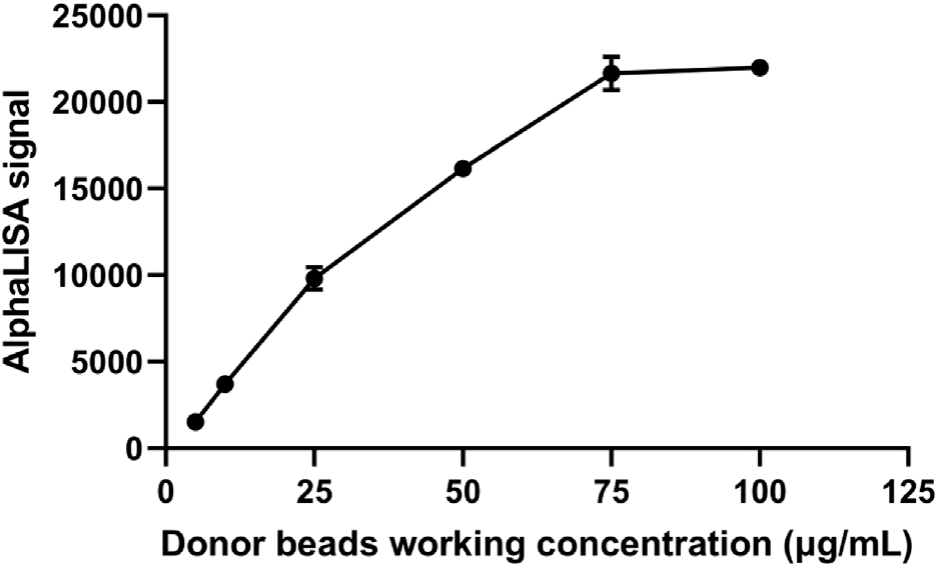
Selection of optimal donor bead concentration.

Previous experiments revealed that when the concentration of the donor and acceptor beads is relatively high, their mixture leads to elevated nonspecific signals. If the concentration of the donor and acceptor beads is too low, then the fluorescence counts are low, which will affect the measurement range. Therefore, we chose 75 μg/mL as the initial fixed concentration that showed a low background and a high binding rate to meet the requirements of sensitivity and measurement. Acceptor beads concentration fixed first, as the concentration of donor beads coupled with capture antibody increased, the AlphaLISA signal showed a significant increase (Figure 2). As the donor beads concentration reached 75 μg/mL, the AlphaLISA signaling uptrend flattened out, suggesting that the donor beads concentration was saturated. Thus, the optimum donor beads concentration for subsequent tests was confirmed to be 75 μg/mL.

### 3.2 Selection of the optimum acceptor beads concentration

**FIGURE 3:**
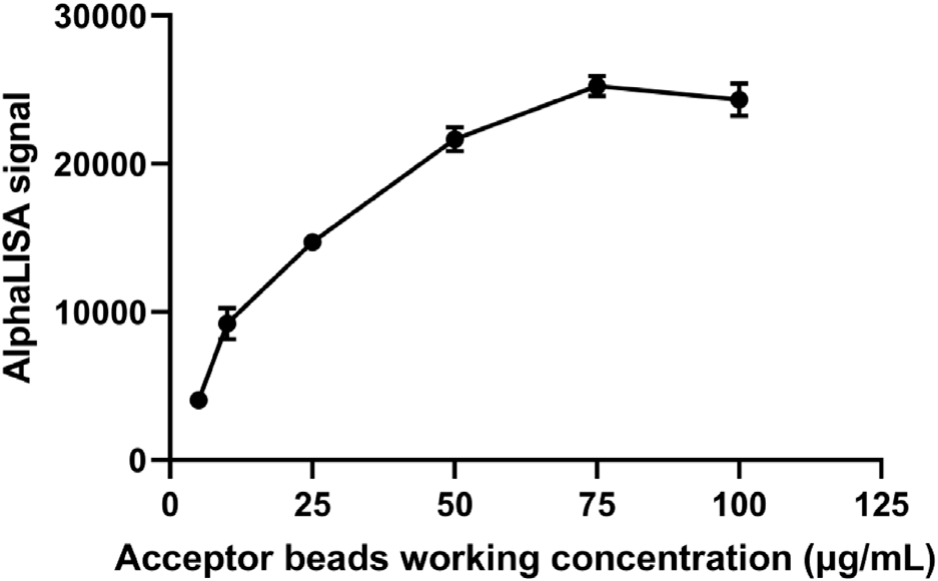
Selection of optimal receptor bead concentration.

The concentration of donor beads was controlled and the concentration of acceptor beads was varied to screen the optimal concentration of acceptor beads. The signal intensity increased with increasing concentration of the acceptor beads (Figure 3). The AlphaLISA signal was stable at the acceptor beads concentration of 75 μg/mL. Therefore, the optimal concentration of acceptor beads for this method was determined to be 75 μg/mL.

### 3.3 Selection of optimal incubation time

**FIGURE 4:**
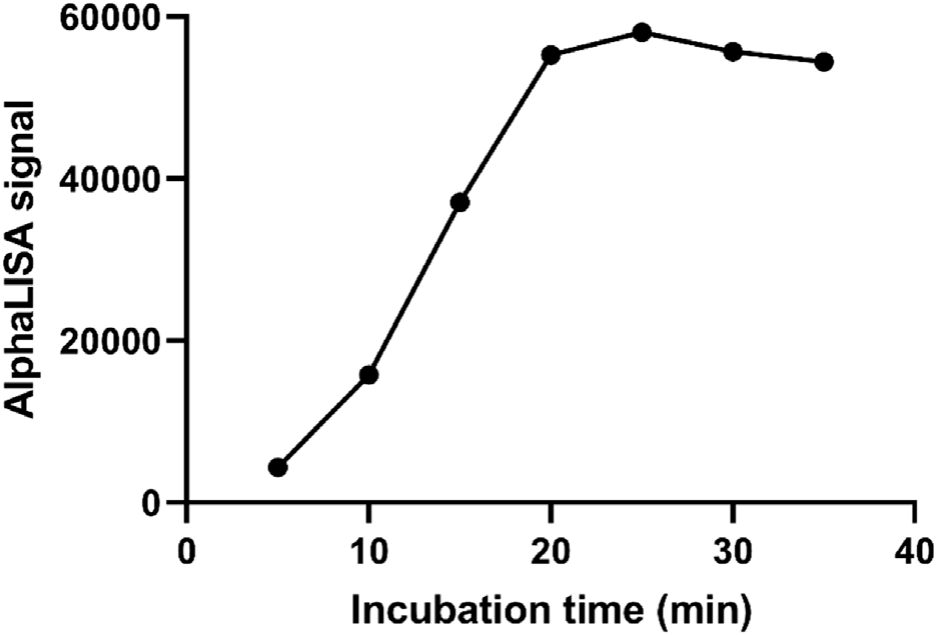
Selection of optimal incubation time.

As demonstrated in Figure 4, the AlphaLISA signal showed an increasing trend with increasing incubation time (5–20 min). When the reaction time reached 25 min, the AlphaLISA signal did not increase significantly and leveled off, indicating that the reaction was close to saturation. Therefore, the optimal incubation time for this method was determined to be 20 min.

### 3.4 Standard curve

**FIGURE 5:**
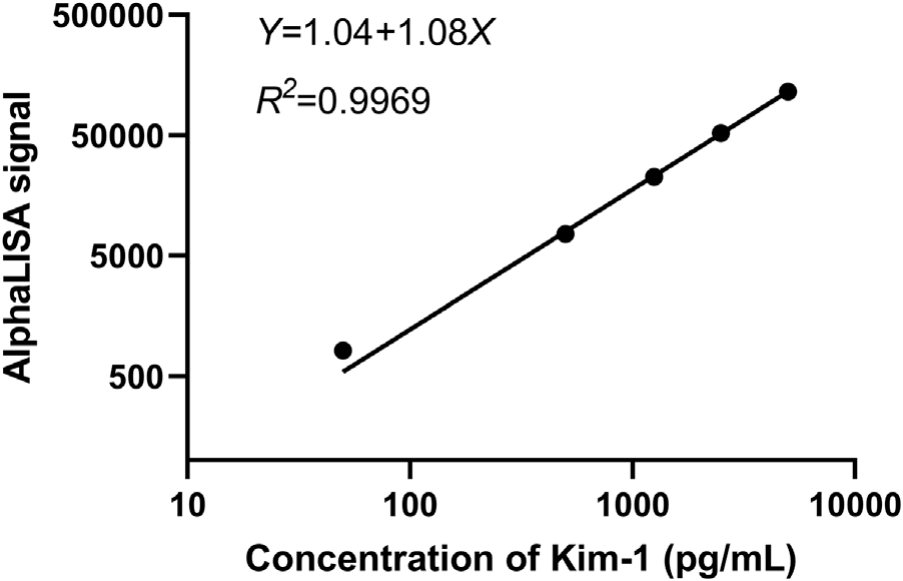
Standard curve of Kim-1.

As shown in Figure 5, the equation of the Kim-1 standard curve is as follows: *Y* = 1.04 + 1.08X (*R* ^2^ = 0.9969), as measured by the Tecan M200 detector under the optimal donor microsphere concentration and the optimal acceptor microsphere concentration, where X represents the sample concentration in pg/mL, and Y represents the AlphaLISA signal of the sample. The result indicated that the standard curve had a good fit. The actual concentration of the sample can be calculated by simply detecting the AlphaLISA signal of the same batch of samples and incorporating it into the standard curve equation of the same batch of experiments. For accurate and consistent experimental results, a standard curve equation must be established for each batch of experiment, and the concentration of each sample must be determined based on the fluorescence signal.

### 3.5 Sensitivity

The sensitivity of the detection method was 3.83 pg/mL, which was calculated by detecting the AlphaLISA signal of the 0 concentration point for 10 times. The detection range of the established detection method was 3.83–5,000 pg/mL.

### 3.6 Precision

As shown in Table 1, the intra-assay coefficient of variation was 3.36%–4.71% (<10%), and the inter-assay coefficient of variation was 5.61%–11.84% (<15%). The results showed that the method exhibited favorable stability.

**TABLE 1:** Kim-1 intra and inter precision.

	Concentration	Average (pg/mL)	Standard deviation	CV(%)
Intra-assay (n = 10)	Low	380.46	17.92	4.71
	Medium	1,033.16	34.69	3.36
	High	2,367.66	86.16	3.64
Inter-assay (n = 10)	Low	349.54	41.41	11.84
	Medium	1,034.94	78.11	7.54
	High	2,348.35	131.76	5.61

### 3.7 Specificity

As shown in Table 2, the cross-reactivity rates of L-FABP (100 ng/mL), NGAL (100 ng/mL), and MMP-3 (500 ng/mL) were 0.0001%, 0.0003%, and 0%, respectively. A very low cross-reaction existed between Kim-1-AlphaLISA and the aforementioned biomarkers.

**TABLE 2:** Cross-reaction rates of L-FABP,NGAL,and MMP-3 correctly present in pdf.

Interferent (pg/mL)	Concentration (pg/mL)	Determined (pg/mL)	Cross-reactivity (%)
Kim-1	2,500	2,447.92	97.92
L-FABP	100,000	0.182	0.0001
NGAL	100,000	35.52	0.0003
MMP-3	500,000	0.00	0.0000

### 3.8 Recovery

As shown in Table 3, the recovery of serum samples was 92.31%–99.58%, which proved the accuracy of the develo ped method.

**TABLE 3:** Recoveries of samples with different concentrations.

Concentration of surmer sample (pg/mL)	Concentration of standerd (pg/mL)	Theoretical concentration (pg/mL)	Measured concentration (pg/mL)	Recovery rate (%)
2047.65	500	1892.89	1881.69	99.41
	2,500	2092.89	2084.06	99.58
	5,000	2,342.89	2,162.81	92.31

### 3.9 Comparison between AlphaLISA and TRFIA

**FIGURE 6:**
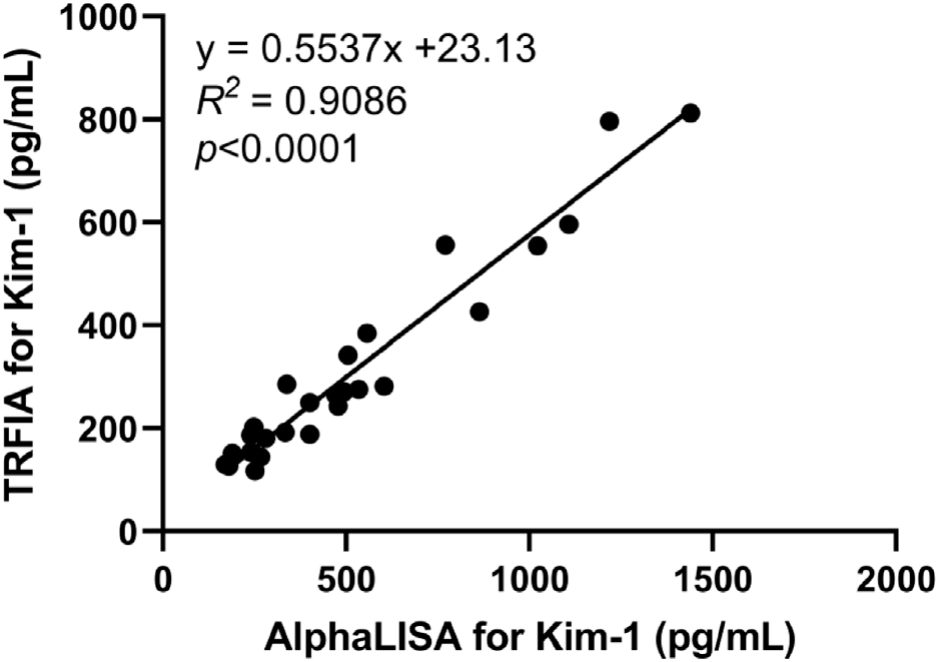
Correlation analysis between the Kim-1-AlphaLISA and Kim-1-TRFIA.

AlphaLISA and TRFIA were used to detect serum Kim-1 concentration in 13 patients with nephropathy and 13 healthy subjects. The two methods had a correlation coefficient *R* ^*2*^ of 0.9086 (Figure 6).

### 3.10 Clinical application of Kim-1-AlphaLISA

**FIGURE 7:**
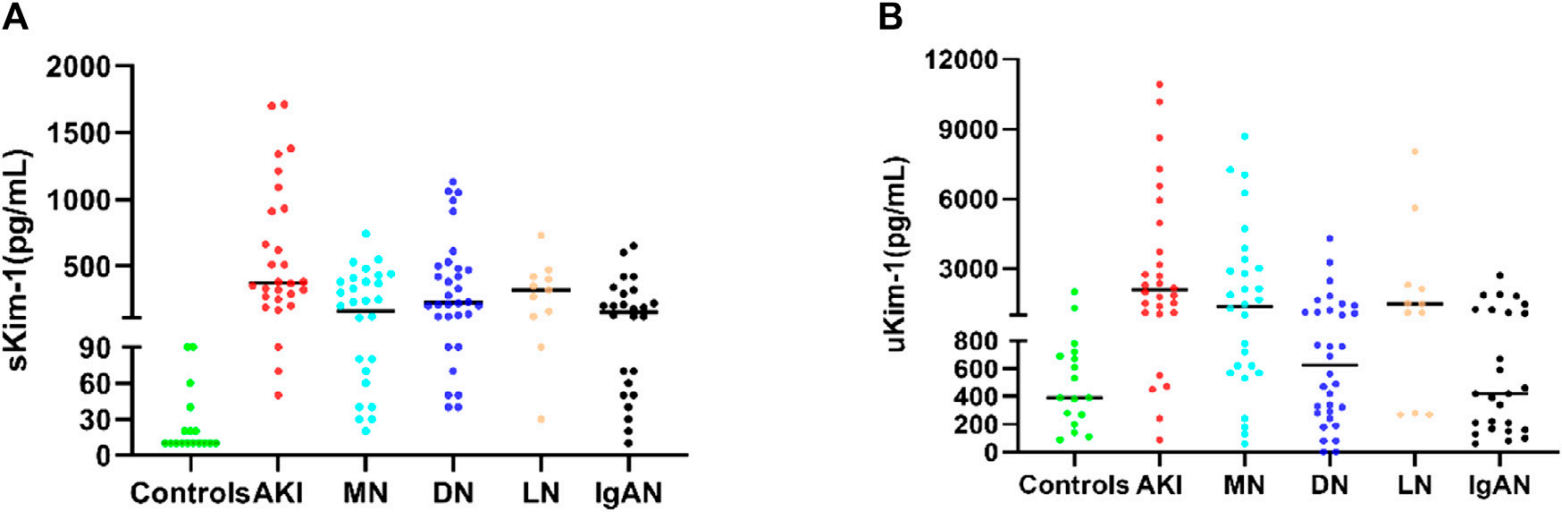
**(A)** Serum Kim-1 concentrations in nephropathic patients and controls. **(B)** Urinary Kim-1 concentrations in patients with nephropathy and controls.

As shown in Figures 7A, B, serum and urinary Kim-1 concentrations were significantly higher in patients with nephropathy than in those in the control group, especially in patients with AKI, whose serum and urinary Kim-1 levels were significantly higher than those of other nephropathies. In addition, serum Kim-1 levels were more favorable than urine Kim-1 levels in distinguishing healthy individuals from patients with nephropathy.

## 4 Discussion

In this study, we developed a new method based on AlphaLISA to rapidly and accurately detect Kim-1 levels in serum and urine. The method is characterized by fast, accurate, and sensitive detection and does not require tedious cleaning steps; as such, it not only reduces the time spent in the experimental process but also avoids the possibility of reducing the immunoassay specificity and sensitivity caused by cleaning. Given that the half-life of the excited singlet oxygen in the donor beads is short at 4 µs and the diffusion range in aqueous solution is limited to about 200 nm, the reaction is confined to a pair of neighboring nanobeads. Thus, the fluorescence signal depends on the antibody–antigen reaction, which binds the donor and acceptor beads to form detectable immune complexes (donor beads and acceptor beads in close proximity <200 nm) (Armstrong et al., 2018). With this microsphere-based approach, the relatively large surface area provided by the nanospheres suspended in the assay solution facilitates the rapid onset of the immune response; coupled with the fact that each donor beads can generate 60,000 single-linear oxygen molecules, such a large surface area can greatly amplify the signal (Li et al., 2018; Xiang et al., 2022), greatly enhanced response sensitivity.

Under the optimized conditions, Kim-1-AlphaLISA established in this study had good analytical sensitivity and linear range (3.83–5,000 pg/mL) and better performance than Kim-1-TRFIA (11–4,666 pg/mL). In previous studies, human Kim-1 levels were generally low (Driver et al., 2014; Liu et al., 2020). Therefore, high sensitivity can play a great role in future research. According to the relevant standards of the Clinical and Laboratory Standards Institute (CLSI), the intra-assay coefficient of variation must be CV<10%, and the inter-assay coefficient of variation must be CV<15%, and the standard range of the recovery rate is between 85% and 115%. Our established AlphaLISA method had a coefficient of variation for the intra-assay batch was 3.36%–4.71% (<10%), that of the inter-assay batch was 5.61%–11.84% (<15%), and the recovery rate was 92.31%–99.58%; these values were within acceptable limits. For clinical applications, the method can ensure the stability of the assay results in each batch of experiment, and its good intra-assay and inter-assay batch coefficients of variation can ensure the consistency of consecutive assay results. In addition, the KIM-1 detection results from this method were consistent with the standard KIM-1 concentrations without any matrix effect, and the results were reliable and can be used for clinical examination. In addition, no cross-reactivity of other nephrotic biomarkers was observed. In previous studies, Kim-1-TRFIA showed a good correlation with Kim-1-ELISA (Shaoxiong et al., 2022); in the present experiment, the results of Kim-1-TRFIA and Kim-AlphaLISA showed a good correlation (*R* ^*2*^ = 0.9086). Thus, the developed method can be used to detect Kim-1 levels in serum and urine in clinical conditions and provide reliable, accurate, and stable results.

In our study, Kim-1 levels in serum and urine were significantly higher in patients with AKI, MN, LN, DN, and IgAN than in those of controls; in particular, they were more pronounced in patients with AKI, which might be associated with more severe kidney injury in patients with AKI. A correlation between Kim-1 concentrations and the severity of renal injury has been reported. In particular, AKI led to inflammation and exacerbated tubular injury, while Kim-1-mediated phagocytosis protected against renal injury by down-regulating inflammatory and innate immune responses in acute ischemic and toxic injuries. However, when Kim-1 is chronically expressed, it can lead to progressive renal fibrosis and chronic renal failure (Yang et al., 2015). Therefore, when Kim-1 concentration in serum or urine is elevated, it suggests that the patient has AKI. Furthermore, it has been reported that compared with uKim-1 concentration, elevated sKim-1 concentration in diabetic patients is more responsive to the decline of renal function in patients with nephropathy with the progression of the disease (Gohda et al., 2020). In our research, we found that serum Kim-1 levels were more useful than urine Kim-1 levels for distinguishing between healthy individuals and patients with kidney disease, thus, elevated serum Kim-1 levels should be taken more seriously in clinical practice. Urine levels may be affected not only by glomerular lesions but also by tubular reabsorption capacity, especially in the presence of tubular dysfunction, where tubular reabsorption capacity is affected and urine Kim-1 levels are altered. These results revealed that Kim-1 is a potential biomarker for the diagnosis of AKI (Wen and Parikh, 2021), both at serum and urine levels. Shao (Shao et al., 2014) et al. estimated the diagnostic sensitivity and specificity of Kim-1 in AKI to be 74% and 86%, respectively. AKI is an increasingly frequent disease as part of acute kidney disease, defined clinically as a rapid loss of kidney function (Liu et al., 2020; Shaoxiong et al., 2022), which can be fatal if not treated promptly (Geng et al., 2021). AKI has been reported to be a common complication in hospitalized and critically ill patients (Vijayan et al., 2021), and it can be induced by a variety of risk factors, such as sepsis, cirrhosis, acute pancreatitis, cancer, chronic kidney disease, COVID-19, and cardiac surgery (Campbell et al., 2014; Gaffney and Sladen, 2015; Nassar and Qunibi, 2019; Gupta et al., 2021; Kuwabara et al., 2022; Wu et al., 2022). These risk factors can lead to the development of AKI with a poor prognosis. Given the risk of AKI, Kim-1 testing technology must be timely, rapid, and accurate, with results available as quickly as possible to avoid exacerbation. The traditional method, ELISA, takes more than 3 h to detect Kim-1 and is not sufficiently sensitive (Chaturvedi et al., 2009). Time-resolved fluorescence immunoassay for Kim-1 was recently developed; although the sensitivity has been greatly improved, the detection time remains long (2 h) (Shaoxiong et al., 2022). Compared with TRFIA, AlphaLISA has the advantages of high sensitivity, high stability, time-saving, sample-saving, and easy operation. For example, TRFIA requires 50 μL of sample. After 80 min of reaction, the sensitivity is 11 pg/mL. By contrast, AlphaLISA requires 25 μL of sample for the experimental, detection can be performed within 20 min of reaction. Colloidal gold-based immunochromatographic strip can be considered for the detection of Kim-1 in urine; it has a short time but low sensitivity (Jin et al., 2017). Patients with acute kidney injury require timely treatment, and a rapid and accurate diagnosis is a prerequisite for timely treatment. However, KIM-1 by detection ELISA and TRFIA usually lasts 1–2 h. One of the objectives of this experiment is to provide a fast and accurate detection method. Yuexing Tu et al. (Tu et al., 2014) reported that Kim-1 in patients with septic AKI was significantly elevated within 6 h upon admission to the ICU, peaked within 24 h, remained significantly elevated for 48 h, and had a mortality rate three times that of non-AKI patients. A mate analysis showed that Kim-1 may be a specific predictor of early AKI in patients undergoing cardiac surgery (SHAO et al., 2014). Another study noted the prognostic value of serum and urine Kim-1 for CKD outcomes (LIU et al., 2022). In addition, it has been hypothesized that the elevation of Kim-1 in AKI complicated by acute pancreatitis is of shorter duration and can only be observed with more frequent monitoring of this marker (Wajda et al., 2020). Considering the risks faced by patients with AKI, rapid and accurate diagnosis is a prerequisite for timely treatment. Related to this, the Kim-1 AlphaLISA method can provide clinicians with rapid and reliable results in 20 min. Furthermore, its excellent sensitivity and precision, coupled with its easy operation, meets the need for the continuous monitoring of patients and mastering of dynamic changes in the condition, which can better meet the clinical needs for rapid Kim-1 detection. In addition, this approach can better meet the clinical demand for rapid and precise Kim-1 assay, this technique greatly improves the diagnostic efficiency and contributing to early and effective treatment.

Limitations: First, because it is a one-step double antibody sandwich reaction, it is more likely to produce a HAMA reaction if heterophilic antibodies are present in the sample, generating false positives or false negatives, which require the addition of more blocking agents to reduce the generation of the HAMA effect. Second, the AlphaLISA assay is a homogeneous reaction system without a wash step. When the antigen concentration is too high, the ratio of antigen–antibody reaction is not appropriate, which leads to a decrease in the number of antigen–antibody immune complexes formed, resulting in false negatives known as the “HOOK effect.” Thus, the method is insufficient for measuring very high concentrations of samples. In our work, Kim-1 concentration in human serum or urine is very low, the high concentration is less than 10,000 pg/mL, and the measurement range of Kim-AlphaLISA that we have established is within the range of 50–5,000 pg/mL. While these can be considered good results, we did not find the HOOK phenomenon when the concentration is within the range of 10,000 pg/mL; thus, such an effect does not have much impact on the clinical detection results of Kim-1. We have added a discussion of the limitations of the Kim-1-AlphaLISA assay in the fourth paragraph of the discussion.

In conclusion, our study established for the first time a rapid and accurate method for the detection of Kim-1 in serum and urine based on AlphaLISA technology. This method exhibits satisfactory performance and can meet the need for rapid and accurate detection in clinical diagnosis, especially in patients with AKI.

## Data Availability

The original contributions presented in the study are included in the article/Supplementary Material, further inquiries can be directed to the corresponding authors.
